# Comparison of a unified analysis approach for family and unrelated samples with the transmission-disequilibrium test to study associations of hypertension in the Framingham Heart Study

**DOI:** 10.1186/1753-6561-3-s7-s22

**Published:** 2009-12-15

**Authors:** Xiangqing Sun, Tao Feng, Yeunjoo Song, Robert C Elston, Xiaofeng Zhu

**Affiliations:** 1 Department of Epidemiology and Biostatistics, Case Western Reserve University, Cleveland, Ohio 44106, USA

## Abstract

Population stratification is one of the major causes of spurious associations in association studies. A unified association approach based on principal-component analysis can overcome the effect of population stratification, as well as make use of both family and unrelated samples combined to increase power (family-case-control, or FamCC). In this study, we compared FamCC and the transmission-disequilibrium test (TDT) using data on hypertension, systolic blood pressure, and diastolic blood pressure in the Framingham Heart Study. Our study indicated FamCC has reasonable type I error for both the unrelated sample and the family sample for all three traits. For these three traits, we found results from FamCC were inconsistent with those from the TDT. We discuss the reasons for this inconsistency. After correcting for multiple tests, we did not detect any significant single-nucleotide polymorphisms by either FamCC or the TDT.

## Background

Population stratification is one of the major causes of spurious associations in association studies. Several approaches have been developed to deal with this problem. The genomic control [1], structured association [2,3], and principal-component analysis methods [4-8] correct for population stratification in population-based case-control studies by using a set of markers across the genome. The transmission-disequilibrium test (TDT) makes use of family structure to match the cases and controls on their genetic background and thus avoids the inflated type I error rate due to population stratification. For a binary trait, it tests association by comparing the frequencies of alleles transmitted and those of alleles not transmitted from heterozygous parents to affected children. A unified association method (family-case-control, or FamCC), which utilizes both unrelated and family samples, was developed based on principal-component analysis [9]. The population background, represented by the principal components, is calculated from a large number of genetic markers typed on unrelated subjects and family members, and then used to adjust the genotype and phenotype values. Because it can make use of both unrelated and family samples, this method uses more information than the TDT. It has no rare disease assumption, while accepting multiple affected and unaffected siblings, which is a limitation of another association method that combines family and unrelated samples [10].

In this study, the unified association method FamCC [9] and the TDT were compared for association tests of the binary trait hypertension and quantitative traits systolic blood pressure (SBP) and diastolic blood pressure (DBP) in the Framingham Heart Study data.

## Methods

### Samples

A total of 13,336 subjects in 1,231 pedigrees are included in the Framingham Heart Study. They are from three generations: the original generation, their offspring, and the third generation. Subjects in the original generation were discarded for this analysis because of concern over the age of their DNA samples. There are 6,395 genotyped and phenotyped subjects in the offspring generation and the third generation, from 1,144 pedigrees, and they were all used as the family sample for this association study. When the original generation was discarded, some large pedigrees were broken, which resulted in 1,705 nuclear families and 1,022 singletons. In order to determine how FamCC would handle a completely unrelated sample, 1,109 biologically unrelated best genotyped individuals with age greater than 20, single founders or founder couples, were taken from the offspring generation of the family sample to form a subsample of unrelated individuals.

### Markers

There were 487,014 single-nucleotide polymorphisms (SNPs) across the genome genotyped for each subject on the Affymetrix 500k chip. In all, 22,775 SNPs on chromosome 9 were used for our association study of blood pressure because of the linkage evidence identified on chromosome 9 in a previous study [11]. After eliminating SNPs with more than 10% missing genotypes, 20,266 SNPs remained. Then the SNPs with minor allele frequency less than 5% or with Hardy-Weinberg equilibrium test *p*-value < 2.47 × 10^-6 ^were dropped, resulting in 15,622 SNPs for the final analysis.

### Blood pressure phenotypes

SBP and DBP were measured for the two cohorts (offspring and generation 3) at four examinations (examination 1, examination 3, examination 5, and examination 7). One binary trait, hypertension, and two quantitative traits, SBP and DBP, were used as the phenotypes in this study. Hypertension was defined as having been treated for hypertension or if, at any of the four examinations, SBP was higher than 140 mm Hg or DBP was higher than 90 mm Hg. For the quantitative SBP and DBP phenotypes, we first added 10 mm Hg to SBP and 5 mm Hg to DBP for individuals on hypertension treatment, as suggested by Tobin et al. [12]. Then for SBP and DPB, adjustments were made for sex, age, BMI, and cohort effects for each examination using multiple linear regression. The average residuals over the four examinations for SBP and DBP were used as the final SBP and DBP phenotypes in the association analyses.

### Statistical methods

FamCC first makes use of principal components to adjust for population stratification, and then tests the null hypothesis of no association. There are three steps in FamCC [9]. In step 1, principal components are generated from the marker data. In step 2, multiple linear regression on the top 10 principal components is performed for both the phenotypes and markers, respectively, to estimate the coefficients in the linear regression models. Because the principal components represent genetic population diversity, linear regression is aimed at adjusting out any population stratification. In the first two steps, all the available unrelated individuals in the data are used. In step 3, the residuals of the phenotypes and the markers for each individual in the data are calculated, and then association between the phenotype and genotype is examined by testing the correlations between these residuals.

In this study, we modified the association statistic in FamCC when we calculated the variance of the correlation between a marker genotype residual and phenotype residual. We considered each family as a random draw from a population and calculated the variance of the correlation between genotype and phenotype among the families. The genotype and phenotype correlation *T*_*k *_for the *k*^th ^family is , where  and  are the residuals of marker genotype and phenotype values for individual *j *in the *k*^th ^family after adjusting for the principal components, *n*_*k *_is the number of individuals in the *k*^th ^*family *(*n*_*k *_= 1 for families of size one, namely, for unrelated individuals, and *n*_*k*_>1 for families with multiple individuals). Define the statistic *T *as

where *N *is the total number of families, and *N*_*u *_is the number of families of size one, that is, the number of unrelated individuals. Thus, the statistic *T *has two parts, the first part is calculated from unrelated individuals and the second part is from families. We calculate the variance of *T *for families and unrelated individuals separately, that is

where  and  are the variances of *T*_*k *_for unrelated individuals and families, respectively. They can be estimated from the sample variance as

Thus, estimating the pair-wise correlation between family members for phenotype and genotype [9] is now unnecessary.

The TDT was conducted with the family-based association testing (FBAT) software [13], using the -e option to test the null hypothesis of no association in the presence of linkage (i.e., the alternative is the presence of both association and linkage), in which the correlation among sibling marker genotypes is adjusted by an empirical variance-covariance estimator [14]. The additive genetic model was assumed.

In this study, FamCC was used to analyze both the whole family sample and the unrelated subsample. Note that although the whole family sample was subjected to the TDT analysis, only the data available in the 1,705 nuclear families could be informative for that analysis.

## Results

**Figure 1:**
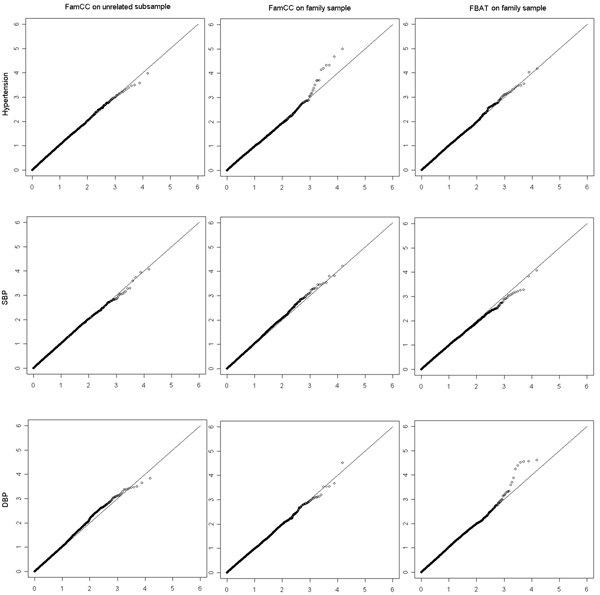
**Q-Q plots of the *p*-value distributions for FamCC and FBAT**. The left column shows the plots from FamCC using the unrelated subsample, the middle column are the plots from FamCC using the family sample, and the right column are the plots from FBAT using the family sample. The three rows are respectively for hypertension, SBP and DBP. x axes, expected distribution; y axes, observed distributions; the scale is -log_10_(*p*).

Figure 1 presents the *p*-value distributions for the SNPs on chromosome 9 by the modified FamCC and the TDT, in which no obvious deviation from the expected null distribution was observed. The inflation factor *λ *[15], estimated by the mean of the test statistic values across all the SNPs, was close to 1 for FamCC for all three traits (Table 1), indicating that FamCC can control the inflation of type I error well. FBAT also has good control of type I error. As shown in Table 1, FamCC had reasonable type I error at the nominal 0.05 level for all three traits, the highest error rate being 0.053 for hypertension using the unrelated subsample. The highest type I error for FBAT was 0.055 for the phenotype DBP.

**Table 1:** Inflation factor and type I error for FamCC and FBAT

Method	Sample	Inflation factor *λ*			Type I error (0.05 level)^a^		
			
		Hypertension	SBP	DBP	Hypertension	SBP	DBP
FamCC	Unrelated subsample	1.03	1.01	1.02	0.053	0.049	0.051
FamCC	Family sample	1.06	1.03	1	0.048	0.051	0.051
FBAT	Family sample	1.00	0.98	1.02	0.051	0.046	0.055

^a^On the assumption that all SNPs with *p*-values < 0.05 represent type I errors

Table 2 presents SNPs identified by FamCC and FBAT with *p*-values < 0.0001.

**Table 2:** SNPs on chromosome 9 identified by FamCC and FBAT with *p*-values < 0.0001

			*p*-value^a^			
				
SNP	Position (bp)	Allele Freq.	FamCC Unrelated subsample	FamCC Family sample	FBAT Family sample	Gene
**Hypertension**						
rs278714	75348574	0.06	0.01	**7.24 × 10^-5^**	0.30	373 kb to *ANXA1*
rs368408	75355214	0.06	0.01	**6.26 × 10^-5^**	0.26	380 kb to *ANXA1*
rs17761685	102914240	0.20	0.08	**4.71 × 10^-5^**	0.40	*PRG-3*
rs17829436	102932851	0.18	0.19	**4.65 × 10^-5^**	0.46	*PRG-3*
rs17829926	102944699	0.19	0.12	**2.12 × 10^-5^**	0.54	*PRG-3*
rs4278208	102969600	0.19	0.11	**1.02 × 10^-5^**	0.52	*PRG-3*
rs2984524	74179463	0.48	0.20	0.30	**6.90 × 10^-5^**	10 kb to *ZFAND5*
**SBP**						
rs4979219	109463841	0.16	**8.52 × 10^-5^**	0.02	0.38	172 kb to *KLF4*
rs10961684	14718052	0.25	0.25	**6.11 × 10^-5^**	0.75	5 kb to *CER1*
rs2583845	132603386	0.29	0.15	0.16	**8.50 × 10^-5^**	*ABL1*
**DBP**						
rs1547761	16908196	0.30	0.08	**3.08 × 10^-5^**^a^	5.00 × 10^-3^	40 kb to *BNC2*
rs7859491	121679109	0.10	0.70	0.10	**5.70 × 10^-5^**	500 kb to *DBC1*, *CDK5RAP2*
rs3934946	121680170	0.10	0.71	0.06	**2.70 × 10^-5^**	500 kb to *DBC1*, *CDK5RAP2*
rs41332646	121688986	0.10	0.90	0.04	**4.10 × 10^-5^**	500 kb to *DBC1*, *CDK5RAP2*
rs10984755	121691216	0.10	0.91	0.04	**3.00 × 10^-5^**	500 kb to *DBC1*, *CDK5RAP2*
rs10984756	121691605	0.10	0.90	0.07	**2.80 × 10^-5^**	500 kb to *DBC1*, *CDK5RAP2*
rs10984758	121691719	0.10	0.73	0.04	**2.40 × 10^-5^**	500 kb to *DBC1*, *CDK5RAP2*

^a^Bold font indicates *p*-value <10^-4^.

For hypertension, FamCC identified six SNPs using the family sample; two of them also had *p*-values = 0.01 by FamCC using the unrelated subsample. However, these SNPs had *p*-values > 0.05 by FBAT. For SBP, FamCC detected SNPs rs4979219 and rs10961684 using the unrelated subsample and the family sample, respectively, and FBAT detected SNP rs2583845. For DBP, FamCC detected one SNP (rs1547761) using the family sample that had a *p*-value 0.005 by FBAT. FBAT identified six SNPs with *p*-values < 0.0001, and three of them had *p*-values < 0.05 by FamCC using the family sample. Most of the SNPs listed in Table 2 are located in gene desert regions. Four SNPs are in linkage disequilibrium (LD) and are located on the gene *PRG-3*, and one is in the gene *ABL1*.

## Discussion

We compared FamCC and the TDT for an association study of hypertension in the Framingham Heart Study data and found a number of interesting results.

The modified FamCC statistic, which does not require estimating the pairwise genotype-phenotype correlations between family members, has reasonable type I error rates for both the family sample and the unrelated subsample. Moreover, on checking the inflation factor with FamCC for each trait, no obvious inflation of type I error was observed. Using FamCC to identify genes associated with hypertension, we found that although there are several SNPs deviating from the expected null distribution (Figure 1), four of these SNPs were in strong LD, suggesting duplication of signals.

Using the same family sample, FamCC and FBAT produced different levels of significance for the same SNP in association testing of the three traits-hypertension, SBP, and DBP. Such differences also arose when comparing FBAT and the generalized estimating equation approach in a study by Levy et al. [16]. These inconsistent results reflect the different information used by these two approaches and the fact that, because of these different assumptions, it is the alternative hypotheses that are different. For FBAT, the alternative hypothesis is one of both linkage and association, while for FamCC it is association only. FBAT, controls for population stratification by comparing transmitted with non-transmitted alleles from heterozygous parents, ignoring the information in homozygous parents, while FamCC applies principal components obtained from marker genotype data to adjust for any stratification in testing the null hypothesis of association only. FamCC uses all the available phenotype and genotype data, therefore the effective sample size for the TDT method is much smaller than that for FamCC, which would be expected to result in higher power for FamCC, as shown by Zhu et al. [9]. Moreover, none of the detected SNPs reached the 0.05 significance level after correcting for multiple tests (corresponding to the nominal *p*-value 2.5 × 10^-6^). Thus, the SNPs identified by both FamCC and FBAT may only reflect the randomness of the *p*-values under the null hypothesis. Because the information used and the alternative hypotheses assumed by FamCC and FBAT are not the same, observing inconsistent *p*-values from the two methods is not surprising.

## Conclusion

In this study, we compared FamCC and the TDT using data on hypertension, SBP, and DBP in the Framingham Heart Study data. Our study indicated FamCC has reasonable type I error. We observed inconsistent results produced by FamCC and the TDT. The inconsistency can be attributable to the fact these two methods are based on different assumptions, and use different information. These results may also reflect randomness of the *p*-values of the two methods under the null hypothesis. However, their performance, especially the power of the modified FamCC and the TDT, should be further investigated by simulation studies.

## List of abbreviations used

DBP: Diastolic blood pressure; FamCC: Family-case-control; FBAT: Family-based association testing; LD: Linkage disequilibrium; SBP: Systolic blood pressure; SNP: Single-nucleotide polymorphism; TDT: Transmission disequilibrium test.

## Competing interests

The authors declare that they have no competing interests.

## Authors' contributions

XS integrated the data and performed the data analyses. TF modified the FamCC program for this study. YS prepared the data from the original Genetic Analysis Workshop 16 data. XZ and RCE conceived of and designed the study. XS, XZ, and RCE drafted the manuscript. RCE, XZ, and XS critically revised the manuscript for important intellectual content.
